# Pitfalls of Antiretroviral Therapy: Current Status and Long-Term CNS Toxicity

**DOI:** 10.3390/biom12070894

**Published:** 2022-06-26

**Authors:** Harrison Rudd, Michal Toborek

**Affiliations:** 1 Department of Biochemistry and Molecular Biology, University of Miami Miller School of Medicine, Miami, FL 33136, USA; harrisonkylerudd@gmail.com; 2 Institute of Physiotherapy and Health Sciences, The Jerzy Kukuczka Academy of Physical Education, 40-065 Katowice, Poland

**Keywords:** HIV, antiretroviral therapy, blood-brain barrier, brain, neuroHIV

## Abstract

HIV can traverse the BBB using a Trojan horse-like mechanism. Hidden within infected immune cells, HIV can infiltrate the highly safeguarded CNS and propagate disease. Once integrated within the host genome, HIV becomes a stable provirus, which can remain dormant, evade detection by the immune system or antiretroviral therapy (ART), and result in rebound viraemia. As ART targets actively replicating HIV, has low BBB penetrance, and exposes patients to long-term toxicity, further investigation into novel therapeutic approaches is required. Viral proteins can be produced by latent HIV, which may play a synergistic role alongside ART in promoting neuroinflammatory pathophysiology. It is believed that the ability to specifically target these proviral reservoirs would be a vital driving force towards a cure for HIV infection. A novel drug design platform, using the in-tandem administration of several therapeutic approaches, can be used to precisely target the various components of HIV infection, ultimately leading to the eradication of active and latent HIV and a functional cure for HIV. The aim of this review is to explore the pitfalls of ART and potential novel therapeutic alternatives.

## 1. Introduction

**Figure 1:**
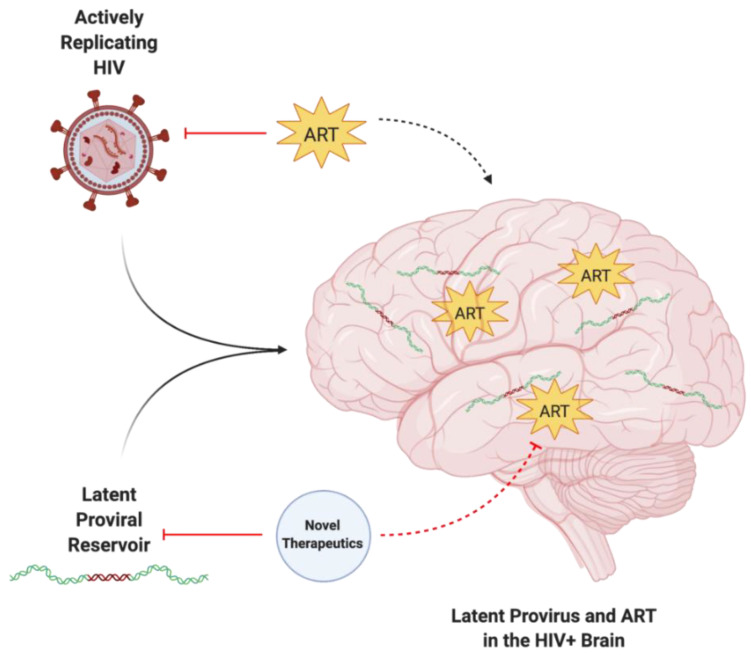
The enigma of persisting latent HIV provirus despite ART. Actively replicating and latent forms of HIV can infiltrate the CNS, resulting in neuroinflammatory pathophysiology. While ART can target active HIV, it is unable to target latent proviral reservoirs. The in-tandem use of ART and novel therapeutic approaches is required to target and eliminate both active and latent HIV within the CNS. Created with BioRender.com. Abbreviations: ART = antiretroviral therapy; HIV = human immunodeficiency virus.

Infection with human immunodeficiency virus (HIV) was once considered to be near-certainly fatal. Approximately 40 million people are currently infected worldwide by HIV, including approximately 2 million children under the age of 15 [1]. Antiretroviral therapy (ART) has changed the landscape of the morbidity and mortality of HIV infection, providing HIV-infected (HIV^+^) individuals with a means to achieve long-term viral suppression within peripheral circulation while also quelling viral activity within the CNS. Most HIV^+^ patients undergo lifelong treatment with ART due to the aggressive nature of HIV infection and the potential for rebound viraemia [2]. Evidence suggests that the antiretroviral drugs (ARVds) commonly used to treat HIV infection can be toxic within the CNS and can result in the development of various pathophysiologies [3,4,5,6,7,8,9,10,11,12,13,14]. While ART can control and inhibit actively replicating HIV, the virus can persist undetected within the host genome in the form of a latent, replication-competent provirus, which can later become reactivated [2,15]. Thus, the current review focuses on pitfalls of ART, including the inability to specifically target the latent HIV provirus, long-term toxic exposure, and limited BBB penetrance, along with novel therapeutic approaches aimed at mitigating these concerns (Figure 1).

## 2. HIV Infection within the CNS

### 2.1. The Blood-Brain Barrier

The blood-brain barrier (BBB) is the crucial anatomic and biochemical interface responsible for regulating the microenvironments between peripheral circulation and the central nervous system (CNS) [16,17,18]. These highly regulated microenvironments are required for neural signaling and the maintenance of homeostasis within the CNS. Additional barriers including the blood–cerebrospinal fluid (CSF) barrier and the arachnoid barrier provide additional supportive functions in maintaining CNS homeostasis, but are not as crucial nor do they occupy as large of a surface area as the BBB. As such, the BBB is at the front line of defense in protecting the highly safeguarded CNS from the entrance of toxins and pathogens, including HIV and medications such as ART, adding an element of challenge to drug discovery and design.

At the BBB, a monolayer of cerebral microvascular endothelial cells (CMECs) forms the framework of capillary walls, which are interlocked by tight junctions (TJs) made up of proteins including claudin-5, occludin, and submembranous zona occludnes-1 (ZO-1). These TJs facilitate the regulation of BBB and CNS homeostasis by linking together CMECs, preventing the passage of many paracellular molecules into the brain parenchyma, while also providing a cytoskeletal matrix of intercellular protein filaments arranged as a series of membranous and submembranous barricades that enable the structural and functional maintenance of barrier integrity. CMECs are peripherally surrounded by a basement membrane (basal lamina), pericytes, astrocytic end-feet, and neurons, which together comprise the neurovascular unit and serve to strengthen barrier function and integrity at the BBB [18]. Pericytes and astrocytes have important roles in maintaining structural integrity at the BBB as they can modulate levels of TJ protein expression and vesicle trafficking in CMECs [19] and contribute to various aspects of CMEC phenotype, including development, proliferation, migration, and survival [17,19]. Interestingly, pericytes are also able to regulate the expression of BBB-specific genes in CMECs, influencing overall BBB integrity [20].

There are several pathways that restrict the entry of drugs into the CNS. Molecules that can diffuse or be transported through the endothelium, including ART, can be actively removed via efflux pumps including P-glycoprotein, multidrug resistance proteins, and organic anion transporters [3]. This provides a challenge for ART in reaching therapeutic concentrations within the CNS, allowing for the possibility of rebound viraemia. It is, however, essential to note that several factors can modulate the expression of these transport proteins, such as inflammatory, genetic, and drug-induced interactions [3], which can result in increased transport of ART across the brain endothelium, leading to increased toxic exposure. Of particular interest is the role of P-glycoprotein in limiting entry into the CNS for ARVds. Substantial effort has been made to dissect the regulatory mechanisms modulating the expression and/or activity of this protein. Exposure of capillaries to low levels of proinflammatory factors, such as lipopolysaccharide (LPS), tumor necrosis factor (TNF)-α, or endothelin-1 (ET-1), was demonstrated to cause a rapid loss of P-glycoprotein transport function with no change in protein expression. On the other hand, a prolonged exposure to proinflammatory factors, including TNF-α, had an opposing effect, i.e., upregulating P-glycoprotein expression via complex mechanisms that shared common signaling elements, such as TNF receptor 1, endothelin receptors, protein kinase C, and nuclear factor-κB (NF-κB) [21]. The role of inflammatory factors in the modulation of P-glycoprotein activity has been confirmed in several literature reports (reviewed in [22]). Interestingly, P-glycoprotein is also involved in the immune inflammatory response in the CNS by regulating microglia activation and mediating immune cell migration [23]. We demonstrated that exposure to HIV-1 Tat protein resulted in overexpression of P-glycoprotein both at mRNA and protein levels in brain endothelial cells and brain microvessels via mechanisms involving NF-κB, intact lipid rafts, and activation of Rho signaling [24,25]. Similar upregulation of P-glycoprotein was demonstrated upon exposure to HIV [26]. In addition, induction of P-glycoprotein in human brain microvessel endothelial cells was demonstrated upon treatment with ARVds via the mechanisms involving human pregnane X receptor (hPXR) and/or human constitutive androstane receptor (hCAR) [27].

Hydrophobicity and low molecular weight are positively correlated with BBB penetration [3,28]; however, efflux pumps may still actively remove these substances from the brain parenchyma. The inability of certain antiretroviral drugs (ARVds) to reach therapeutic concentrations within the CNS may play a role in the potential for rebound viraemia. Thus, future methods of drug delivery must be investigated and optimized to bypass the classical diffusion and transport mechanisms of ART across the BBB.

### 2.2. A Trojan Horse Mechanism for HIV Infection of the CNS

HIV attacks the immune system by infecting and eliminating cells that express the CD4 receptor (CD4^+^ cells) and coreceptors, including the C-C motif receptor 5 (CCR5) and C-X-C motif receptor 4 (CXCR4). The HIV genome can integrate into the host genome of many cell types; however, there are two major cellular reservoirs: CD4^+^ T lymphocytes and macrophages. CD4^+^ T cells are crucial for combating infection and maintaining immune responses, homeostasis, and memory, and as such, are associated with several inflammatory and autoimmune diseases [29]. Macrophages are derived from monocytes and myeloid cells of hematopoietic origin [23]. In the CNS, microglia and partially pericytes are cells of myeloid origin. These cells, along with astrocytes, can all be directly infected by HIV [30,31,32,33,34]. Indeed, a 12 h incubation period with two strains of HIV resulted in the cellular entry of HIV and low-level replication of HIV in human brain pericytes, astrocytes, and CMECs [35].

**Figure 2:**
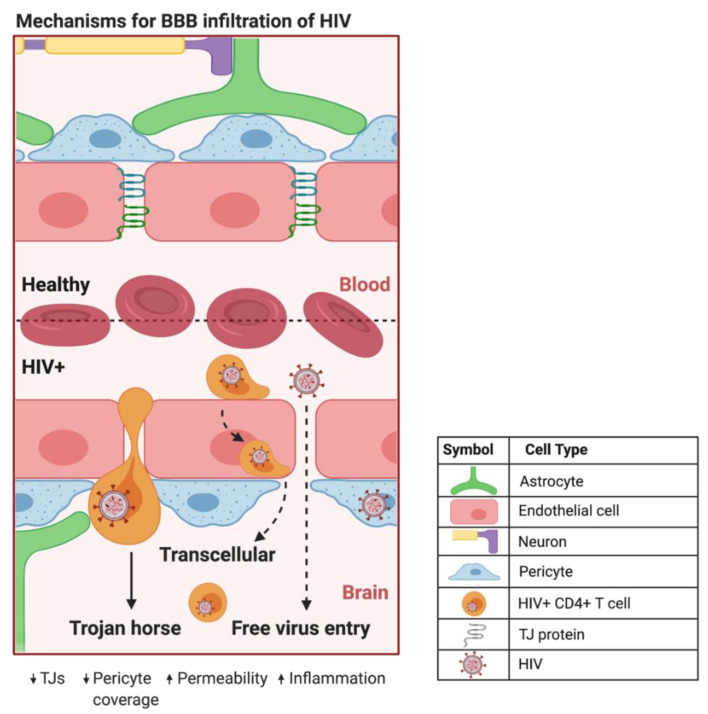
Proposed mechanisms of BBB infiltration of HIV. At the HIV^+^ BBB, infected CD4^+^ T cells and monocytes can cross by several proposed mechanisms. The predominate method centers around HIV using infected CD4^+^ T cells and monocytes as a Trojan horse to paracellularly infiltrate brain parenchyma. HIV^+^ monocytes can also transcellularly pass through CMECs at the BBB. As HIV infection progresses in the CNS, increased BBB permeability and decreased expression of TJ proteins can provide a pathway for HIV to paracellularly invade the brain parenchyma. Created with BioRender.com. Abbreviations: HIV^+^ = human immunodeficiency virus-infected; TJs = tight junctions.

HIV can infiltrate the CNS early in the course of infection. HIV evades detection by the immune system primarily by using HIV^+^ CD4^+^ T cells and cells of the monocytic lineage in a Trojan horse approach to traverse the BBB [35] (Figure 2). The free virus is also able to cross the BBB through TJ openings that can result from HIV-induced dysfunction of CMECs [16]. In addition, HIV^+^ pericytes were shown to stimulate dysregulation of BBB integrity via decreased TJ protein expression [34]. This HIV-induced increase in BBB permeability can lead to the activation of microglial cells and uncontrolled migration of immune cells into the CNS, which are capable of causing neuroinflammation, loss of neural tissue, and infection due to the influx of pathogens [36]. In addition, studies have shown that CMECs can undergo apoptosis during HIV infection [16,34], which increases BBB permeability and can promote the infiltration of HIV^+^ cells and virions into the CNS. Intriguingly, HIV-specific proteins, such as transactivator of transcription (Tat), are capable of inducing CMEC dysfunction and subsequent BBB dysregulation [37,38,39], enhancing the infiltration of HIV across the BBB. The entry of HIV into the CNS can result in neuropathological dysfunctions ranging from sub-clinical and minor cognitive impairments or motor deficits to HIV-associated neurocognitive disorders (HAND), including dementia [40]. ART administration is negatively correlated to TJ protein expression and BBB permeability [19,36,41], illustrating the need for drug design to maximize the efficiency of BBB crossing and to overcome toxicities associated with the administration of ART.

### 2.3. Elusive Latent Proviral Reservoirs within the CNS

**Figure 3:**
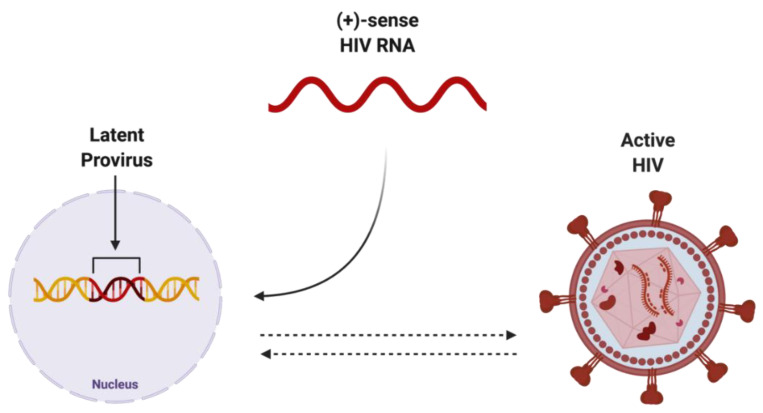
Potential endpoints of positive-sense HIV RNA after integration into host genome. Once integrated into the host genome, (+)-sense HIV RNA can persist as either latent provirus, which is capable of being reactivated, or actively replicating HIV, which can be deactivated. Created with BioRender.com. Abbreviations: (+)-sense = positive-sense; HIV = human immunodeficiency virus; RNA = ribonucleic acid.

The integration of reverse-transcribed viral DNA into the host genome is a crucial step in propagating both the active and dormant forms of HIV (Figure 3). Once integrated, the proviral DNA serves as the transcriptionally competent viral unit and the central source of viral protein production. The gene expression of HIV is controlled by promoter and enhancer sequences where transcription factors, including NF-κB, can bind, promoting RNA polymerase II activity, ultimately resulting in increased virus-specific protein levels [15]. Transcriptional inactivity of the HIV proviral DNA results in the latent proviral stage of HIV, where the virus can remain dormant in the host genome as a transcriptionally incompetent reservoir for later reactivation.

**Figure 4:**
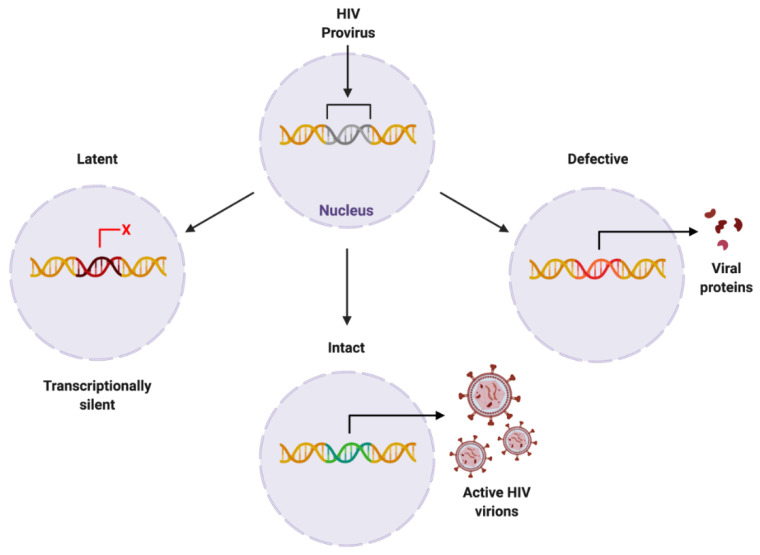
Proposed forms of HIV proviral reservoir. HIV provirus can persist in three forms: latent, being transcriptionally silent; intact, producing active HIV virions; or defective, containing genetic mutations resulting in viral protein synthesis. Created with BioRender.com. Abbreviations: HIV = human immunodeficiency virus.

The HIV provirus can exist in three forms: latent, which is transcriptionally silent; intact, producing active virions; or defective, producing viral proteins but not able to successfully replicate [42] (Figure 4). Intact and latent HIV proviral reservoirs have the potential to cause rebound viraemia, whereas defective provirus does not. It is important to note that while defective HIV provirus is not replication-competent, these malfunctioning viral DNA sequences can produce viral HIV proteins, which can propagate pathogenesis. Furthermore, cells latently infected with HIV can release exosomes containing viral mRNA and protein, hijacking intercellular communication networks as a means to reactivate latent reservoirs, transmit infection, and further disease development [43], presenting another target required to fully eradicate infection with HIV.

HIV can exist as a latent proviral reservoir in several cell types, namely CD4^+^ T cells and cells of monocytic lineage. Cells that are latently infected with HIV provirus can evade detection by the immune system and may be replicated via the homeostatic proliferation of their host cell [42]. Although microglial cells are the primary reservoir cell type within the CNS [44], there is novel evidence depicting astrocytes and pericytes as constituents of these dormant HIV cellular reservoirs [31,32]. For example, integrated viral DNA has been discovered in microglia and pericytes within the CNS tissue of post-mortem HIV^+^ patients [30,42], illustrating the likelihood of myeloid-derived reservoir sites within the brain. Intriguingly, novel research indicates a key role for neurons, as opposed to non-neuronal cell lines, in the stimulation of HIV latency in microglia [45]. In addition, neurons can prevent the emergence of active HIV from latency and neuronal damage can induce replication and activation of HIV [45]. As cells of the myeloid lineage are long-lasting and recent investigation has illustrated both inductive and preventative roles for neurons in HIV latency, it is crucial to further investigate the functional properties of HIV latency.

Activating the transcriptionally silent, latent HIV proviral reservoir can be achieved with the use of histone deacetylase inhibitors. Histone deacetylase inhibitors promote the acetylation of histones and consequential chromatin relaxation, facilitating the accessibility of transcription factors to DNA and enabling transcription of the viral genome via RNA polymerase II recruitment. For example, pericytes in the latent stage of HIV infection that were treated with histone deacetylase inhibitors and tumor necrosis factor (TNF) exhibited a significant increase in HIV-1 RNA and HIV p24 protein production, illustrating that pericytes can alternate between the latent and active viral stages [30]. The mechanisms underlying HIV proviral transcriptional silencing and reactivation are not yet fully understood. Recent investigation has revealed a method of measuring and discerning the intact versus defective proviral HIV genome [46], which is a crucial step toward curing HIV infection. Specific targeting of the latent proviral reservoir remains the central obstacle in achieving complete viral eradication from HIV^+^ individuals.

Perivascular spaces in the CNS also contain populations of cells capable of harboring HIV. In a macaque model, perivascular macrophages and microglia were shown to harbor SIV genomes, which could be reactivated, even after observed antiretroviral therapy suppression [47]. While there is still debate regarding the role that macrophages play in active viral reservoirs of HIV, findings in mice confirm the possible importance of this cell type. Indeed, studies indicated that HIV persists in humanized myeloid-only mice independent of other possible reservoir-capable cell types, such as T-cells, supporting the role of macrophages in HIV replication and formation of viral reservoirs. This mouse model is generated by transplanting CD34^+^ hematopoietic stem cells into immunodeficient nonobese diabetic/severe combined immunodeficiency (NOD/SCID) mice, which are characterized by an absence of functional T and B cells [48,49]. Furthermore, there is mounting evidence that macrophages play an important role in their susceptibility to HIV even after ART initiation (reviewed in [28]).

Evidence indicates that viral entry can occur through the choroid plexus [50,51]. It is well-known that resident macrophages, i.e., the cells that frequently become infected with HIV in the CNS, can line the epithelium of the choroid plexus [52]. As a separate dynamic reservoir for HIV accumulation, the choroid plexus provides a possible path for neuro-invasion events and a conduit for future ART drug delivery. It should also be noted that HIV trafficking via the choroid plexus barrier is coordinated by the high amount of multidrug resistance proteins and P-glycoprotein expressed on the surface [53]. Paradoxically, the P-glycoprotein pump is oriented in a way that opposes the action of P-glycoprotein efflux transporter located in the BBB, whereby it prevents substrates and other molecules from escaping the CSF. This complex relationship further accentuates CNS and BBB homeostasis in trafficking therapeutics to the CNS (reviewed in [28]).

## 3. Pitfalls of ART: Focus on the CNS

There are many shortcomings associated with ART, the current standard therapeutic approach in treating HIV infection. While ART has provided HIV^+^ patients with a means to control the actively replicating virus and decrease patient mortality rates, there are still several key issues that must be overcome when administering ARVds, e.g., limited BBB penetrance, toxic exposure, and the inability to target latent proviral HIV reservoirs, particularly in the CNS. A drug’s ability to traverse the BBB is dependent on an array of factors, including molecular size, polarity, protein–protein intractability, and physicochemical properties [54]. Moreover, drug-induced modulation of transport protein expression may increase the penetrability of brain endothelium to ART and, in turn, expose the CNS to a higher level of toxicity [54]. Taken together, alternative routes of delivery and mechanisms of CNS entry need to be further explored to achieve concentrations sufficient for optimizing the therapeutic effects of ART while minimizing any toxic side effects.

### 3.1. ART Does Not Protect against Latent HIV Infections

**Figure 5:**
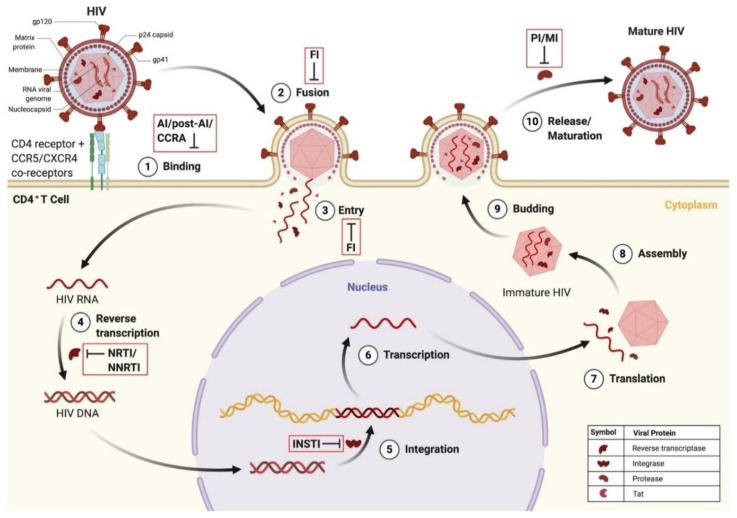
HIV infection of CD4^+^ T cell and points of ARVd intervention in the HIV replication cycle. (1) HIV binds to the CD4 receptor and CCR5/CXCR4 co-receptors. This can be blocked by attachment inhibitors, post-attachment inhibitors, and CCR antagonists. (2) Fusion of the HIV and host cellular membrane occurs. This can be blocked by fusion inhibitors. (3) Entry of viral proteins into the host cell. This can be blocked by fusion inhibitors. (4) Reverse transcription of HIV RNA into proviral HIV DNA. This can be blocked by nucleoside/non-nucleoside reverse transcriptase inhibitors. (5) Integration of HIV DNA into the host genome. This can be blocked by integrase strand transfer inhibitors. (6) Transcription of HIV RNA. (7) Translation of HIV RNA into viral proteins. (8) Assembly of immature HIV. (9) Budding of immature HIV into the host cell membrane. (10) Release and maturation of HIV. This can be blocked by protease inhibitors and maturation inhibitors. Created with BioRender.com. Abbreviations: ARVd = antiretroviral drug; CD4 = cluster of differentiation 4; CCR5 = C-C motif receptor 5; CXCR4 = C-X-C motif receptor 4; DNA = deoxyribonucleic acid; FI = fusion inhibitor; HIV = human immunodeficiency virus; INSTI = integrase strand transfer inhibitor; Tat = transactivator of transcription.

Latent proviral HIV reservoirs are established early in the course of infection and cannot be targeted by traditional therapeutic approaches, specifically ART, making them a primary obstacle in attaining a cure for HIV infection [54]. ART does, however, have the capability to target several stages within the viral replication cycle of HIV (Table 1) (Figure 5). Though this is sufficient to suppress levels of actively replicating HIV and maintain viraemia, latent HIV provirus does not actively replicate and therefore is not a therapeutic target of ARVds. Additionally, the limited BBB penetrance of traditional HIV therapeutics presents a significant obstacle in not only delivering effective doses, which are required for maintaining suppression of viraemia within the CNS in particular, but also in designing novel therapeutics to target latent HIV proviral reservoirs within the CNS.

Despite several years of ART treatment, HIV provirus can persist within the chromosomal DNA of latently infected CD4^+^ T cells [28]. These potent proviral reservoirs are established within hours of infection and are extremely stable, having an average half-life of approximately 44 months [55,56]. Currently, virologic suppression of HIV infection has been defined as having a viral load of fewer than 20 copies per milliliter [57], which can be achieved through lifelong adherence to an ART regimen. Interestingly, many HIV^+^ patients who have successfully gained control of the infection via ART experience intermittent episodes of detectable viraemia (blips) [58], which have been shown to be correlated to HIV reservoir size [59]. Specifically, HIV^+^ patients undergoing treatment regimens with protease inhibitor-based combination ART (cART) have exhibited a higher degree of residual viraemia [57], indicating the inability of ART to target the HIV provirus. In one study, a majority of the simian immunodeficiency virus (SIV)-infected (SIV^+^)^,^ ART-suppressed macaques contained latently infected brain macrophages [60]. Remarkably, latent HIV provirus can persist even when viral RNA is not detectable [60]. Taken together, further investigation is required to elucidate and understand the mechanisms underlying latent HIV proviral persistence despite ART.

### 3.2. ART Has Limited BBB Penetrance

The ability of ARVds to infiltrate the CNS has been hierarchically categorized by CNS penetration efficiency (CPE), which ranges from low (i.e., CPE of 1) to high (i.e., CPE of 4) [36] (see Table 1). When jointly administered, ART can have a cumulative CPE score varying from low (i.e., CPE < 8) to high (i.e., CPE > 8) [29]. Several chemical and physiological properties determine the ability of ARVds to cross the BBB, including pharmacodynamics, pharmacokinetics, and structural characteristics of the drugs (e.g., lipophilic or ligand-receptor interactions), which can alter their half-life and biodistribution. There is a debate centered around what combination of ARVds is ideal for overcoming the challenge of BBB penetrance and optimizing the therapeutic benefits while minimizing potential toxicities associated with ART. ARVds with a higher CPE score (i.e., better CNS penetration) may be advantageous for regulating HIV infection within the brain; however, these drugs may also result in a higher level of toxic exposure as they are able to reach higher levels in the brain [61]. Additionally, the limited ability of ARVds to penetrate the BBB may permit low levels of active HIV replication, potentially increasing latent reservoir size and the chance of rebound viraemia.

### 3.3. ART-Induced CNS Toxicity

Long-term administration of ARVds places patients at an increased risk of toxic exposure, which can result in neurodegeneration, inflammation, and co-/multi-morbidities such as cardiovascular, metabolic, and neurological diseases [40,54,61,62,63,64,65,66,67,68,69,70,71]. The comorbidities and long-term toxic exposure associated with ART can lead HIV^+^ patients to switch or discontinue their medication regimens, ultimately resulting in rebound viraemia. For example, the use of ART has been shown to impact various aspects of cellular function within the CNS (see Table 2), in particular, inducing neurotoxic effects [71] and reducing the viability of endothelial cells exposed to ART even at relatively low concentrations [2]. ART-induced vascular toxicity can result in decreased TJ protein expression and BBB dysregulation [3]. Primary CMECs that were treated with Efavirenz exhibited decreased claudin-5 expression and localization to the cellular membrane, which was attenuated via the inhibition of endoplasmic reticulum (ER) stress prior to ART exposure [36]. Notably, it was found that the use of ART induced oxidative damage and mitochondrial dysfunction in endothelial cells [3,11,12,41,72] and neurons [4,71,73] (see Table 2). These ART-mediated effects stimulate the induction of inflammasomes and lead to an increase in inflammatory cytokines [10,74,75], which can result in a loss of BBB integrity and increased permeability via decreased TJ protein expression [76]. 

**Table 2:** Known effects of ART on cells of the CNS.

Cell Type	Impact from ART	References
Astrocyte	↓ Mitochondria function and metabolism ↓ MMPs ↑ Senescence ↑ ER stress	[3,13,77]
Endothelial Cell	↓ Viability ↓ Mitochondria function ↓ Autophagosome formation ↓ TJPs ↑ ROS production ↑ ER stress ↑ Inflammatory cytokine production	[3,12,36,75,78,79]
Microglial Cell	↓ Lysosomal function ↓ Autophagosomal function ↑ ROS production ↑ Expression of pro-inflammatory cytokines	[10]
Neuron	↓ Axonal length ↓ Neurogenesis ↑ Neuronal death ↑ Oxidative stress ↑ ROS accumulation	[4,73,80,81]
Neural Progenitor Cell	↓ Cell proliferation ↓ Mitochondrial function ↑ Senescence ↑ ROS production ↑ MMP production	[82,83,84]
Oligodendrocyte	↓ Maturation ↑ ROS production ↑ Oxidative stress ↑ Lysosomal stress	[85,86]
Pericyte	↓ Coverage	[87]

Abbreviations: ER = endoplasmic reticulum; MMP = matrix metalloproteinase; ROS = reactive oxygen species; TJPs = tight junction proteins.

The use of ART has been linked to mitochondrial toxicity [3,6,8,9,11,88,89] and oxidative stress [3,4,8,9,11,72]. ART-induced mitochondrial dysfunction may be explained, at least in part, by genetic or epigenetic modulations to mtDNA [74], which impair mitochondrial function and can increase oxidative stress. In particular, protease inhibitors and nucleoside reverse transcriptase inhibitors (NRTIs) are known to induce mitochondrial damage [3,4,70,90,91,92,93]. Exposure to NRTIs has been shown to reduce levels of mtDNA and dysregulate the mitochondrial proteome via the inhibition of polymerase-γ [3,90,92,93]. ART-treated mice exhibited modulated expression of mitochondrial transcription factor A (TFAM) [6]. TFAM occupies roles in the regulation of mitochondrial biogenesis and well as protecting mtDNA [6,94]. A decrease in TFAM observed in neurons of ARV-treated mice supports the notion that mitochondrial biogenesis may be dysregulated by ART. Additionally, NRTIs have been shown to inhibit telomerase, a crucial reverse transcriptase in charge of the de novo production of repeats in telomeric DNA, at therapeutic concentration [92,93]. The inhibition of telomerase can result in downstream interferences in mtDNA replication [93], which can lead to increased mitochondrial aging [91,92] and, ultimately, BBB dysfunction.

Mitochondria are the major source of reactive oxygen species (ROS) production in most mammalian cells, which can occur via the dysregulation of complexes I, II, and III [95,96,97]. Complex I is responsible for the oxidation of nicotinamide adenine dinucleotide (NADH), a crucial step in the electron transport chain, which has been shown to be inhibited in a dose-dependent manner by ARVds [3]. The inhibition and concomitant dysregulation of complex I by ART illustrate a possible mechanism for ART-mediated increase in ROS levels. Protease inhibitors are known to increase oxidative stress through the impairment of mitochondrial function, resulting in mitochondrial damage [4,89,91]. ROS provides a common point of activation for many downstream signaling pathways, which can directly mediate BBB function [98,99], TJ modification [98,99,100,101], and matrix metalloproteinase activation [102]. Therefore, ROS are potentially key mediators in the breakdown and dysfunction of the BBB in ART-treated HIV^+^ patients. Exposure to ART was shown to increase the production of nitric oxide, which resulted in subsequent inflammation [3]. This may be particularly damaging to BBB integrity as nitric oxide synthase activation results in BBB breakdown [103].

Interestingly, a novel study published by our group hints at the metabolic function of certain tight junction proteins, including occludin [104,105], which may result in crosstalk between metabolic function and BBB permeability. Specifically, occludin has NADH oxidase enzymatic activity, which regulates the expression and stimulation of the histone deacetylase sirtuin 1 (SIRT-1). As histone deacetylase inhibitors activate the latent HIV provirus, histone deacetylase, therefore, inhibits latent proviral activation, resulting in a negative correlation between occludin and HIV transcription. In fact, the silencing of occludin in various HIV^+^ cell types resulted in a significant increase in viral transcription via SIRT-1 activation [104]. Thus, further investigation is required to elucidate the signaling pathways tethering metabolic, transcriptional regulation, and BBB permeability functionality of TJ proteins, specifically in the context of ART.

The degradation, clearance, and removal of proteins from cells is a crucial process that converges on three primary organelles: the endoplasmic reticulum (ER), autophagosome, and the lysosome. The exposure of CMECs to ARVds was shown to induce ER stress and autophagy disruption [78], which has been associated with cellular dysfunction and increased permeability in several disease models [36]. For example, ART-treated CMECs exhibited a significant decrease in the expression of the secreted form of alkaline phosphate, which is an indication of ER stress. The elevated expression of stress-indicating ER proteins, including inositol-requiring enzyme 1a and pancreatic ER eukaryotic initiation factor 2a (eIF2a) kinase (PERK), further confirmed ER stress in ART-treated CMECs [78]. PERK was also shown to be upregulated in ART-treated astrocytes [76]. Downstream mediators of these stress-indicating ER proteins were also altered by ART exposure in CMECs [78]. Specifically, increased phosphorylation of eIF2a, which is carried out by PERK, was shown in ART-treated CMECs [78,106] and astrocytes [77]. This effect can result in the decline of a cell’s ability to withstand stressful insults by leading to a reduction in de novo protein synthesis [107].

Autolysosomes are formed from the fusion of autophagosomes and lysosomes and ultimately ensure the completion of autophagy and clearance of misfolded or aggregated proteins [108]. Autophagy is known to be influenced by ER stress and has been shown to be impacted by ART exposure [36]. Light chain 3B (LC3B) is a commonly utilized marker of autophagic function, which is processed from LC3BI (16kDa) to LC3BII (14kDa) upon autophagic activation [78]. Exposure of CMECs to ART was shown to result in decreased processing from LC3BI to LC3BII [78], indicating an ART-induced decrease in autophagic activity. ART was shown to interrupt the maturation stage of autophagy by impairing lysosomal function, ultimately inducing defects in autophagosome-lysosome fusion. Specifically, ART-treated microglia were shown to exhibit impaired lysosomal functioning, which resulted in the accumulation of autophagosomes [10]. In addition, ART was shown to stimulate lysosomal membrane permeabilization, decreased levels of lysosomal-associated membrane protein 2 [10], lysosomal deacidification [10,85], and decreased expression of cathepsin D [10], all of which contribute to lysosomal-mediated autophagy dysfunction and the activation of microglial cells. Interestingly, heat shock protein family A was shown to attenuate ART-mediated lysosomal impairments [10], illustrating promise in moderating the toxic effects associated with ARVds.

It is important to note that the ART-induced products of mitochondrial, ER, and autolysosomal stress can result in the induction of inflammasomes, including the nucleotide-binding oligomerization domain (NOD)-like receptor protein 3 (NLRP3) inflammasome [109,110], which produces inflammatory cytokines such as interleukin-1b (IL-1b) and IL-18, whose discharge by caspase-1 mediates apoptosis [111,112], suggesting a possible means for ART-induced neuroinflammation. The NLRP3 inflammatory pathway responds to the presence of damage-associated molecular patterns (DAMPs), which are produced as a result of ART-induced organelle stress [113]. Remarkably, ART-mediated apoptosis was shown in endothelial cells, which was measured by upregulation in the expression of the pro-apoptotic cytokine pro-caspase-3 [73], which serves an important role in cell death. Furthermore, time-dependent ART-mediated upregulation in the mRNA of pro-inflammatory cytokines IL-1β, IL-6, and TNF was recently discovered in microglial cells [10]. Exposure to ART was also reported to increase in Aβ peptides [114,115] and Aβ deposition [116], as shown in the CSF of HIV^+^ individuals compared to those not utilizing the therapy [115]. These results correspond to elevated levels of Aβ in HIV+ individuals [114,115,116] and warrant further investigations on the impact of ART on cognitive functions and/or accentuated aging frequently observed in HIV^+^ patients [117].

## 4. Novel Strategies in Developing HIV Therapeutics

It is largely agreed that the eradication of HIV is dependent on the ability to target and eliminate both the latent viral reservoir and persistent low-level replication of active virions or inhibit infection with HIV altogether. This demands novel approaches, such as an effective vaccine, allogenic hematopoietic stem cell transplantation (allo-HSCT), gene therapy, and nanotherapeutics, or even a combination of these approaches. The tandem deployment of these novel therapeutic approaches can also serve to mitigate the long-term toxicity observed in ART-treated HIV^+^ patients. 

### 4.1. CNS Targeting of Latent HIV Provirus

Targeting the latent HIV proviral reservoir has been attempted by using a “shock and kill” paradigm, which works by inducing the activation of HIV from latency (shock) and then eliminating HIV viral reservoir cells (kill) [118,119,120]. The kill phase of this paradigm primarily centers around the immunologic elimination of viral reservoir cell sites. As transcription factors such as NF-kB are known to induce HIV replication [15,121], activators of this pathway have been used to shock latent HIV reservoirs into a reactivated state [47,122]. Another approach utilized an activator of the cytokine IL-15 [123] as a shock mechanism, which has been shown to activate the transcription of HIV [124]. This strategy can be combined with the suppression of immune components that have an apparent, albeit unknown, role in the stabilization of HIV latency [124]. It is important to note that while NF-kB activation is a promising shock strategy to reactivate latent HIV reservoirs, inducers of this pathway have low efficacy in acting as latency-reversal agents, often leading to toxic exposure that restricts the clinical application of this potential therapeutic approach [125]. In addition, the reactivation of latent HIV alone is not sufficient to decrease the size of the HIV reservoir, and the kill phase of this paradigm primarily centers around the immunologic elimination of viral reservoir cell sites, which requires optimization [126].

A more recent “block-and-lock” paradigm has provided an alternative promise into a possible method for eradication of the latent HIV proviral reservoir. The “shock-and-kill” paradigm aims to entirely eradicate latent proviral reservoir sites, whereas the “block-and-lock” method is deployed to permanently silence all HIV proviruses, even after the termination of ART [126]. Permanent silencing of the HIV genome can be accomplished by targeting different components of the transcriptional machinery, including the use of a Tat inhibitor to silence the transcription and reactivation of HIV [126]. Indeed, it was shown that treatment with the Tat inhibitor didehydro-cortistatin A (dCA) was able to delay and reduce rebound viraemia in mice [127]. Other elements of viral transcription that can be targeted and inhibited in the “block-and-lock” paradigm include heat shock protein 90 (HSP90) [118], Janus kinus-signal transducer and activator of transcription (Jak-STAT) [119,120], mammalian target of Rapamycin (mTOR) [128], p21-activated kinase (PAK) [129], rapidly accelerated fibrosarcoma kinase (Raf) [129], and bromodomain-containing protein 4 (BRD4) [130], among several other proposed targets of HIV transcription.

Additional approaches for targeting latent HIV proviral reservoirs predominately revolve around gene-therapy methods, including clustered regularly interspaced short palindromic repeats-associated protein nuclease-9 (CRISPR/Cas-9) [15,131,132,133], broadly neutralizing antibodies (bNAbs) [134], and transcription activator-like effector nucleases (TALENs) [135]. CRISPR/Cas9-based systems have been used to precisely target latent HIV proviral DNA [15,136]. Similarly, TALENs have been utilized to accurately target latent HIV DNA, being more effective and having less off-target editing than CRISPR/Cas9-based systems [135]. Interestingly, the use of bNAbs in mice was shown to obstruct the development of the latent HIV proviral reservoir [134]. In fact, consistent with data from human and macaque studies, mice treated with bNAbs 4 days post-HIV infection exhibited viremia approximately 34% less often, which took longer to establish than in mice treated with ART [118]. Major advantages of CRISPR, TALEN, and bNAb-based platforms, besides their ability to specifically target latent HIV proviral DNA, are their pliability in design for individual target sites, high editing capabilities, and minimal toxic exposure [135].

### 4.2. Obstructing Infection by HIV

Along with precisely targeting the latent HIV provirus, the inhibition of infection by HIV has proven possible with the use of novel techniques such as allo-HSCT [137,138]. Allo-HSCT is focused predominantly on a homozygous deletion (CCR5D32/D32) within the C-C motif receptor 5 (CCR5) coreceptor, which is a crucial mediator in the cellular entry and subsequent infection of HIV. Indeed, CD4^+^ T cells lacking this receptor exhibit impaired binding to HIV, ultimately inhibiting downstream viral infection and providing resistance to HIV [139]. There have been reported cases of a functional, ART-free possible HIV cure by using allo-HSCT [137,138,140], including the Berlin patient. In a similar case of allo-HSCT, the Essen patient, rebound viraemia quickly occurred after the interruption of ART [130]. It was determined that the Essen patient had been co-infected with an alternative, pre-existing HIV variant, albeit at low levels, able to transmit the infection through the C-X-C motif receptor 4 (CXCR4) coreceptor [141]. This type of CXCR4 tropic switch may occur later in the course of HIV infection [142,143] and presents a significant challenge that needs to be considered in therapeutic design strategies, specifically regarding the use of gene therapy to cure HIV infection. In addition, risks including graft-versus-host interactions may occur [142].

## 5. Concluding Comments

While ART has provided HIV^+^ patients with a means to control the infection and live relatively normal lives, there are still several pitfalls and shortcomings that need to be further improved and investigated. For example, ART cannot target or eradicate the latent HIV provirus, ultimately failing to cure infection with HIV. A long-term regimen of ART places HIV^+^ patients at the risk of developing toxicity and the subsequent domino effect of pathogenesis, specifically in the CNS. In addition, ARVds have limited BBB penetrance, hindering delivery to the CNS, and failing to target another crucial component within the landscape of HIV infection. On the plus side, ART has the capability of targeting various elements within the HIV replication cycle of active virions, which is necessary to suppress viraemia.

**Figure 6:**
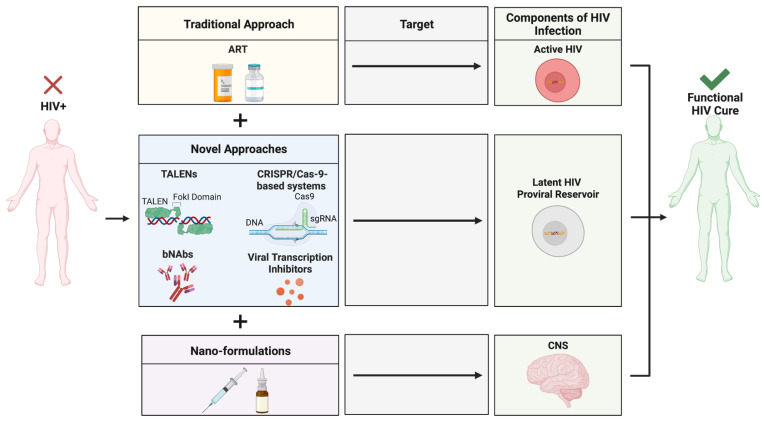
Novel approaches for the eradication of HIV within the CNS. A novel, multimodal drug delivery platform for anti-HIV therapeutics can result in the discovery of a functional HIV cure, allowing for the eradication of actively replicating HIV, latent proviral reservoirs, and specific targeting of the highly safeguarded CNS. Created with BioRender.com. Abbreviations: ART = antiretroviral therapy; bNAbs = broadly neutralizing antibodies; CNS = central nervous system; CRISPR/Cas-9 = clustered regularly interspaced short palindromic repeats-associated protein nuclease-9; HIV = human immunodeficiency virus; TALENs = transcription activator-like effector nucleases.

A multimodal approach utilizing the in-tandem administration of several novel anti-HIV therapies may be an effective strategy in designing a cure for HIV infection (Figure 6). The use of these novel approaches in concert may prove to be less toxic and/or more effective than the use of ART alone [135,144]. A recent study demonstrated the use of magneto-electric nanoparticles bound with a CRISPR/Cas-9-based system to cross the BBB and inhibit latent HIV infection in microglial cells [131]. Additional studies have investigated the use of nano-formulations developed for the delivery of ART [145,146,147] and combining ART with mitochondria-targeted antioxidant therapy [148]. Specifically, nano-based drug-delivery systems have been developed for the delivery of tenofovir [146], and graphene quantum dot-based systems have been used to inhibit HIV replication similarly to NNRTIs [147]. As the investigation continues to deepen our understanding of the crucial elements that may provide systemic eradication of HIV infection, it is clear that new avenues of therapeutic innovation are required. Taken together, the use of these modern approaches illustrates the promise of novel therapeutic strategies in augmenting traditional ART, providing a higher degree of targeting efficiency and ultimately curing HIV infection.

## Figures and Tables

**Table 1:** Antiretroviral drug classes, function, examples, and dosage reference.

ARVd Class	Function	Drug Examples	CPE Score	Side Effects	Adult Dosage Schedule
Nucleoside reverse transcriptase inhibitor (NRTI)	Inhibits reverse transcriptase, blocking production of viral DNA	Lamivudine (3TC)	2	Nausea, dizziness, lactic acidosis, pancreatitis, IRIS	300 mg once or 150 mg twice daily
		Zidovudine (ZDV)	4	Nausea, dizziness, lactic acidosis, liver problems, myopathy, severe anemia, neutropenia, IRIS, lipoatrophy	250–300 mg twice daily
		Emtricitabine (FTC)	3	Nausea, dizziness, lactic acidosis, IRIS, possible HBV flare up	200 mg daily
		Tenofovir (TFV)	1	Nausea, dizziness, lactic acidosis, kidney problems including kidney failure	300 mg daily
Non-nucleoside reverse transcriptase inhibitors (NNRTI)	Binds to and blocks HIV reverse transcriptase, blocking production of viral DNA	Efavirenz (EFV)	3	Nausea, dizziness, mental health problems, liver problems, severe rash, nervous system issues, seizures, IRIS, lipodystrophy, hyperlipidemia	600 mg daily with a NRTI or PI
		Nevirapine (NVP)	4	Nausea, dizziness, severe liver problems, skin rash, IRIS, lipodystrophy syndrome	200 mg twice daily
Fusion inhibitors (FI)	Inhibits viral binding or fusion of HIV to host target cells preventing the entry of HIV	Albuvirtide * (ABT)	ND	Nausea, headache, diarrhea, rashes, hyperlipidemia	ND
		Enfuvirtide (T20)	1	Allergic reaction, nausea, headache, pneumonia, neuralgia, IRIS	90 mg twice daily
Protease inhibitors (PI)	Blocks proteases required for proteolytic cleavage of precursors necessary viral replication	Atazanavir (ATV)	2	Nausea, dizziness, heart arrhythmia, severe rash, liver problems, life-threatening drug interaction, chronic kidney disease, kidney stones, gallbladder problems, IRIS, lipodystrophy, increased bleeding in hemophiliacs, diabetes, and hyperglycemia	300 mg with 100 mg RTV daily
		Darunavir (DRV)	3	Nausea, dizziness, liver problems sever skin reactions, diabetes, hyperglycemia, lipodystrophy, IRIS	600–800 mg with 100 mg RTV daily
		Ritonavir (RTV) ^⊥^	1	Nausea, dizziness, pancreatitis, heart arrhythmia, severe allergic reactions, liver problems, hyperlipidemia, hyperglycemia, IRIS, lipodystrophy, increased bleeding in hemophiliacs, gastrointestinal problems	600 mg twice daily
Integrase strand transfer inhibitors (INSTI)	Prevents the integration of HIV DNA into host DNA	Dolutegravir (DTG)	ND	Nausea, dizziness, allergic reactions, liver problems, IRIS, sleep problems	50 mg once or twice daily
		Raltegravir (RAL)	3	Nausea, dizziness, severe skin reactions, allergic reactions, liver problems, IRIS	1200 mg daily or 400–800 mg twice daily
Chemokine coreceptor antagonists	Blocks coreceptors (CCR5/CXCR4) preventing the entry of HIV	Maraviroc (MVC)—CCR5	3	Nausea, dizziness, liver problems, heart problems (including heart attack), skin reactions, allergic reactions, postural hypotension, IRIS, possible increased risk of other infections or cancer	150 mg, 300 mg, or 600 mg twice daily depending on concomitant medications
		Leronlimab * (PA14)	ND	Diarrhea, headache, swollen lymph nodes, hypertension, local injection site reactions	ND
CD4 attachment inhibitors/post-attachment inhibitors	Binds to host CD4 receptor blocking HIV attachment and entry	Ibalizumab-uiyk (IBA)	ND	Nausea, dizziness, IRIS, diarrhea, rashes	Loading dose of 2000 mg and maintenance doses of 800 mg every two weeks
		UB-421 * (mAb dB4)	ND	Rash, hives, increased eosinophil count, elevated liver enzyme levels	ND

Abbreviations: CPE = CNS penetration efficiency; CCR5 = C-C motif chemokine receptor 5; CXCR4 = C-X-C motif receptor 4; HBV = hepatitis B virus; HIV = human immunodeficiency virus; mAb = monoclonal antibody; mg = milligram; ND = not yet determined; TAG = triacyglyceride; IRIS = immune reconstitution inflammatory syndrome. * = In clinical trials. ^⊥^ = Used clinically as pharmacokinetic enhancer to inhibit metabolism of CYP3A enzymes and increase bioavailability of PIs.

## Data Availability

Not applicable.
